# Ninth Annual Announcement of Medical Department Iowa University

**Published:** 1858-06

**Authors:** 


### NINTH ANNUAL ANNOUNCEMENT

OF THE

### College of Physicians and Surgeons,

OF THE

### IOWA STATE UNIVERSITY.

**BOARD OF TRUSTEES:**

Hon. MATURIN L. FISHER, Pres't of the Board, ex-of.
" ANSON HART, Secretary, Iowa City.
His Excel. RALPH P. LOWE, Trustee, ex-officio.

| | |
|---|---|
| Hon. GEO. W. McLEARY, Iowa City, | Terms expire in 1858. |
| " ANSON HART, " " | |
| " E. C. LYON, " " | |
| " JAS. H. GOWER, " " | |
| " G. D. PALMER, " " | |
| " EDW. CONNELLY, " " | Terms expire in 1859. |
| " H. W. LATHROP, " " | |
| " M. J. MORSMAN, " " | |
| " J. W. RANKIN, Keokuk. | |
| " P. L. LAKE, Bellevue, | Terms expire in 1861. |
| " LAURIN DEWEY, Mt. Pleasant, | |
| " T. FARMER, M'Kissack's Grove, | |
| " E. C. BIDWELL Quasqueton, | |
| " AMOS WITTER, Davenport, | |

Hon. LINCOLN CLARK, (to fill vacancy), Dubuque, term expires in 1859.

**CURATORS:**

Hon. George G. Wright, Chief Justice of the Supreme Court.
" W. S. Woodward, Associate " " "
" L. D. Stockton, " " " "

| | | |
|---|---|---|
| Hon. Hugh T. Reid, | E. H. Harrison, Esq., | Col. Wm. Thompson, |
| E. R. Ford, M. D., | Hosmer Curtis, | Col. C. Perry, |
| Wm. Leighton, | Rev. W. H. Williams, | D. W. Kilbourne, Esq. |
| John M. Hyatt, | Edw. Kilbourne, Esq., | Hon. Charles Mason, |
| James L. Estes, | Samuel F. Miller, Esq. | Hon. Hawkins Taylor, |
| V. P. Van Antwerp, | Hon. R. P. Lowe, | Charles Parsons, Esq., |
| Rev. Prof. E. Gunn, | Rev. W. H. Cowles, | D. W. Ford, Esq., |
| Hon. James M. Love, | Col. Wm. Patterson, | A. L. Deming, Esq. |

### MEDICAL DEPARTMENT.

### AMOS DEAN, L. L. D.,

PRESIDENT OF THE UNIVERSITY.

### FACULTY.

JNO. R. ALLEN, M. D.,

Emeritus Professor of Obstetrics.

D. L. McGUGIN, M. D.,

Professor of Physiology and Pathology, and President of the Faculty.

FREEMAN KNOWLES, M. D.,

Professor of Obstetrics and Diseases of Women and Children.

J. C. HUGHES, M. D.,

Professor of Surgery and Dean of the Faculty.

A. S. HUDSON, M. D.,

Professor of Principles and Practice of Medicine.

WELLS R. MARSH, M. D.,

Professor of Chemistry and Toxicology, and Treasurer of the Faculty.

DANIEL MEEKER, M. D.,

Professor of Anatomy.

Professor of Materia Medica, Therapeutics and Microscopy, (to be supplied.)

HENRY STRONG, A. M.,

Lecturer on Medical Jurisprudence.

D. C. DEWEY, M. D.,

Demonstrator of Anatomy.

J. WARD DAVIDSON, M. D.,

Prosector of the Chair of Anatomy.

### Announcement.

Notwithstanding the stringency of the times, and the wide spread financial embarrassments, the class of last winter was large and the number of graduates as usual. Those upon whom the Degree of "Doctor of Medicine" was conferred were well prepared at their final examination, a fact as creditable to them as it was gratifying to the faculty. We wish it distinctly understood that qualification alone will entitle those who may apply for the highest honors in the profession. The faculty would prefer giving a blank list than pass upon the undeserving in order to present a full catalogue of graduates. We aim to make medical scholars, rather than display, well knowing that such a course will be fully appreciated by the profession. No consideration will induce us to depart from this rule. The profession is assured that there is no diminution of zeal, on the part of the Faculty and Trustees, in furthering the great objects and designs of the institution; and, that every effort will be exerted to favor the progress of the students in acquirement, and in qualifying them for usefulness, thereby elevating the standard of the profession.

### Free Lectures.

The American idea of making the education of its citizens one of the first duties of governments, it is well known, has been carried out in all of the new States by donation to them by the general government of school and university lands. The proceeds of these lands are made a permanent endowment to common schools and a university, the interest of which is devoted to the maintenance of teachers and faculties in the same.

In the original organization of the medical department of the Iowa State University this consideration was given its due weight with the avowed intention of carrying out to the full extent the idea so soon as the pecuniary condition of the institution should seem to warrant the step. In view of this, some years since, a system of scholarships, to be placed in the hands of certain state officers, and by them conferred upon students seeking instruction in the State Institution, was duly arranged and carried out. These scholarships entitled the holder to instruction in the institution at a price considerably reduced from that of those who were not in receipt of them. In this manner the benefits of the institution have been extended to many who must otherwise have been forced to forego them.

But the time having arrived when it seemed possible to carry out fully the original intentions, the Trustees have concluded to announce the *entire abolition of fees*, except those for matriculation and graduation. In doing this they feel that they are but consenting to a demand of the public sentiment both within and without the profession, and that demand one in whose justice they heartily concur.

In view, however, of the reduction of expense of education to the student, it is the determination of the Trustees and Faculty to demand in return full compliance with the established requisitions, and furthermore it will be the constant aim of those concerned in the management of the institution to assist in elevating the standard of these requirements until they shall be able to secure the unqualified approbation of the profession of the country.

---

**University Buildings, Apparatus, &c.**

The college buildings heretofore occupied having been found insufficient for the accommodation of the classes, a new brick edifice is in process of erection, and will be completed at the opening of the ensuing session at a cost of $25,000. This building, sixty-five by ninety feet, and three stories high, will contain two spacious lecture rooms, a commodious laboratory, dissecting room, museum and hospital room sufficient for thirty to forty beds.

Arrangements have also been made for the purchase of new appliances for teaching the various departments. The chemical laboratory will be replenished with new apparatus sufficient, with that already in possession, to make the facilities for teaching this science all that can be desired.

In practical anatomy the advantages are ample. The supply of material is abundant, and every student is required to pursue practical dissection for at least one session. The Demonstrator will be in constant attendance during the hours devoted to this department in the dissecting room, and twice each week will give brief and familiar lectures on some of the most important subjects connected with this science.

There is, connected with this department in the University, a museum, already valuable and yearly becoming more so. It includes numerous valuable and accurate botanical plates, a great variety of casts and models, including many wax preparations, a splendid suit of Materia Medica, abundant dry and wet anatomical preparations and morbid specimens, with many interesting illustrations of natural history, &c., &c.

To increase the number of surgical operations and induce patients from a distance to present themselves, all necessary arrangements have been made for their accommodation by the professor of surgery.

*All Surgical operations before the class are performed gratuitously.*

---

### Hospital and Dispensary.

The University Hospital was constructed by the city, in immediate connection with the old college buildings, and was placed under the medical control of the faculty. It will be so arranged that students may enjoy equally all its advantages. Clinical instruction will be regularly given by the appropriate professor at this hospital.

In connection with the new college building, the faculty are about to establish a city dispensary for the purpose of securing further clinical instruction to the students, and every pains will be taken to afford opportunity for each to personally examine the cases prescribed for.

In addition to the above advantages, the "County Alms House" will be open for the admission of students once a week for clinical instruction, as well as to witness surgical operations. This institution contains at this time about fifty beds, and the number will be increased by the time the session opens.

**Course of Lectures---Session of 1858-9.**

The regular course of lectures will commence on Thursday, the 4th day of November, and continue four months. Six lectures will be delivered daily on the various branches of medical science. The dedication of the new College edifice, and Introductory to the course, will be delivered on Thursday evening. The annual graduation exercises will be held, and the degrees conferred at the close of the term. Every student will be required, within two weeks after the opening of the session to take his ticket and pay the regular fees.

The following are the requisites for the diploma:

1. The candidate must be twenty-one years of age—and present testimonials of a good moral character.

2. He must have attended two full courses of medical lectures; one of which must have been delivered in the medical department of the Iowa State University; or evidence of three years reputable practice will be regarded as equivalent to one course.

3. The candidate must have studied medicine three years, (including the course of lectures) under the direction of a respectable medical practitioner.

4. He must furnish an original medical Thesis, (in his own handwriting, accompanied with his graduation fee), which must be handed to the Dean at least four weeks before the close of the session, and which shall, upon examination prove satisfactory.

5. He must, at the close of the term, pass an examination satisfactorily to the faculty.

Fees, &c.—Matriculation ticket $10 00; the fee for the Diploma is $30 00; the fee for admission into the dissecting room and demonstrations is $5 00. Each student at the time of matriculating must deposit with the Dean $2 00 for the purpose of defraying expense of repairing any damage done by the class to the buildings or appliances. Such damages will be assessed at the close of the term, and the balance of money after paying for the same will be refunded to the student.

Members of the profession from every part of the country, who are graduates of medicine, will, on presenting their diploma to the Dean, be admitted gratuitously to all the lectures.

Board can be obtained in the city at from $2 50 to $3 50. Students, upon arriving in the city, can obtain information as to good boarding houses, by calling at the office of the Dean, on Second street, near the St. Charles Hotel.

Medical books may be purchased in the book stores in our city, on as good terms as in any western city.

All letters may be addressed to

J. C. HUGHES, DEAN OF THE FACULTY.

### Graduates---Session 1857-8.

| NAME. | RESIDENCE. | SUBJECT OF THESIS. |
|---|---|---|
| MAHLON BAILEY, | Iowa, | Effects and Nature of Medicine. |
| R. J. BELL, | Missouri, | Hepatitis. |
| S. L. COMER, | Illinois, | Gun Shot Wounds. |
| E. C. DAVIS, | Missouri, | Acute Rheumatism, |
| J. Q. EGGELSTON, | Indiana, | Mercury and some of its Preparations. |
| H. M. HALL, | Illinois, | Scarlatina. |
| J. A. F. HOLLOWAY, | Maryland, | Yellow Fever. |
| A. C. LITTLE, | Illinois, | Typhoid Fever. |
| E. D. LOMBARD, | Wisconsin, | Typhoid Fever. |
| W. S. MCMICHAN, | Iowa, | Pathological Character, Nature and Causes of Phthisis Pulmonalis. |
| M. S. MCMAHAN, | " | Duties of an Accoucheur in Parturition. |
| R. S. MCKEE, | Missouri, | Colitis. |
| HENRY OSBORNE, | Iowa, | Diagnosis of the Diseases of the Stomach. |
| W. R. PAXTON, | " | Inflammation. |
| MATT. SAVILLE, | Nebras. Ter. | Scurvy. |
| D. W. STOVALL, | Missouri, | Duties of the Accoucher. |
| BENJ. C. TOLER, | Illinois, | Phthisis Pulmonalis. |
| A. M. WALTON, | Indiana, | Absorption of Medicines. |
| W. H. WILMOTT, | Illinois, | Hepatitis. |
| J. A. WOOD, | Indiana, | Nature of Diseases of the Nerves. |

Whole number of Matriculants, - - - - - 75
" " Graduates, - - - - - - 20

### Notices.

To practitioners and students of medicine, who may have received scholarships, we would say that their presentation to the Dean, at the opening of the session, becomes unnecessary, as all students are received upon the payment of the matriculation fee of $10 00.

The faculty would call the attention of the profession, throughout the entire west, to the fact that all patients, medical or surgical, when coming before the class, will be operated upon or prescribed for gratuitously; and the profession would confer a favor upon the institution, and those connected with it, by sending cases which they may not wish to treat themselves. Our surgical cliniques have generally been large—as many as three cases of Lithotomy have been operated upon before the class during one session.

### Text Books.

Students would do well to furnish themselves with copies of some of the following works on the different departments.

ANATOMY.—Morton; Wilson; Cruveilhier.

CHEMISTRY.—Rand; Turner; Lehmann.

INSTITUTES.—Kirkes; Carpenter; Williams.

PRACTICE.—Wood; Watson.

MATERIA MEDICA—Wood's Therapeutics; U. S. Dispensary.

SURGERY.—Miller; Chelius; Erichson; Druitt.

OBSTETRICS.—Cazeau; Meigs; Ramsbotham; Churchill.

DISEASES OF WOMEN.—Colombot; Churchill; Bennett.

DISEASES OF CHILDREN.—Billard; Condie; Meigs.

PRACTICAL ANATOMY—Agnew; Dublin Dissecter; Wilson's Dissector.

MEDICAL JURISPRUDENCE.—Dean; Beck; Taylor.

### IOWA MEDICAL JOURNAL.

J. C. HUGHES { EDITORS. } WELLS R. MARSH.

This is the only Medical Journal published in the State. It is a work of 80 pages, and issued quarterly, for the sum of $2 00—invariably in advance. We are always thankful for subscribers and contributions.

